# The Decrease of Soil Microbial Community Diversity and Network Complexity Results in the Increase of Soil‐Borne Diseases With Monocultural Years in Greenhouse Tomato Production Systems

**DOI:** 10.1111/1758-2229.70165

**Published:** 2025-07-24

**Authors:** Jing Hu, Li Wan, Yafang Wang, Kuai Dai, Klaus Butterbach‐Bahl, Shan Lin

**Affiliations:** ^1^ College of Resources and Environmental Sciences, China Agricultural University Beijing China; ^2^ Sanya Institute of China Agricultural University Sanya China; ^3^ Research Center of Biological Control of Diseases and Insect Pests Engineering of China Tobacco Yuxi China; ^4^ Pioneer Center for Research in Sustainable Agricultural Futures (Land‐CRAFT), Department of Agroecology Aarhus University Aarhus Denmark

**Keywords:** antagonistic bacteria, greenhouse vegetable field, monocultivation years, plant pathogens, soil microbial community

## Abstract

The excessive use of water and fertiliser, along with long‐term monocultivation in greenhouse vegetable fields, has led to a decrease in soil quality and an imbalance in the soil microflora, which may contribute to worsening soil‐borne diseases. To examine the associations between the soil microbial community composition and disease‐suppressing microorganisms under different years of cultivation shifting from croplands to greenhouses, we collected soil samples from fields continuously planted with tomatoes for 5, 10 and 20 years, as well as from nearby maize–wheat fields (MW). The soil physicochemical properties, microbial community composition, putative plant pathogens and putative antagonistic bacteria were analysed. The results indicated that (1) the diversity and richness of soil bacteria and fungi significantly decreased with longer cultivation years; (2) bacterial and fungal diversity indices were negatively associated with soil nutrient content and positively associated with soil pH and NO_3_^−^‐N content, the latter being identified as the main factor contributing to the decline in microbial diversity; (3) the complexity of the soil bacterial network initially increased, peaking after 5 years of continuous monoculture, and then decreased, whereas fungal network complexity gradually declined over time; (4) the relative abundance of putative pathogens such as *Fusarium*, *Alternaria* and *Cladosporium* (associated with tomato wilt and leaf mould) increased significantly with longer cultivation, whereas the relative abundance of the bacterial genera associated with putative antagonism *Bacillus*, *Paenibacillus* and *Streptomyces* decreased. In conclusion, after 10 years of continuous monocultivation, a marked reduction in soil microbial diversity and putative antagonistic bacteria was observed, along with an increase in putative pathogenic fungi. These changes likely contributed to the worsening of soil‐borne diseases, threatening the sustainability of greenhouse vegetable production.

## Graphical abstract

This study highlights the negative impacts of prolonged cultivation on soil microbial diversity, network complexity and the abundance of beneficial microorganisms.

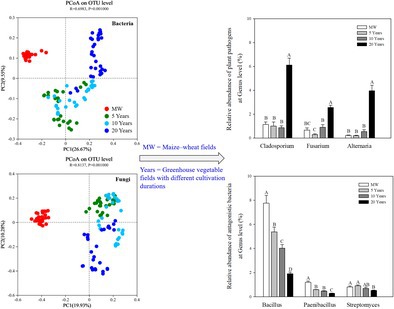

## 1 Introduction

Intensive greenhouse vegetable production has become increasingly important in meeting the global demand for vegetables, not only to support the growing population but also to ensure a stable supply during the winter season (Aznar‐Sánchez et al. 2020). This technology has been adopted by more than 35 countries worldwide for growing vegetable crops (Badji et al. 2022). As the largest producer and consumer of vegetables, China had a vegetable planting area of 22.5 million hectares and a vegetable production area of 791 million tons in 2023. Approximately one‐third of this production came from vegetables grown in greenhouses, especially in the so‐called sunken solar greenhouses in northern China (National Bureau of Statistics of China 2023).

Sunken solar greenhouses are constructed by removing topsoil rich in organic matter to build wind‐break walls, thereby retaining natural warmth in winter without additional heating. The subsoil with lower organic matter content serves as the planting soil layer, which fails to meet the high nutrient and water requirements for the rapid growth of greenhouse vegetables (Lv et al. 2019; Wan et al. 2023). Therefore, low soil fertility, shallow rooting and flood irrigation have driven nitrogen (N) application rates in greenhouse vegetable fields as high as 2500 kg N ha^−1^ year^−1^, roughly five times the crop demand (Lv et al. 2019; Hu et al. 2021; Wang et al. 2022). This not only results in resource waste but also contributes to significant nitrate pollution in groundwater, increased atmospheric N_2_O emissions and soil acidification (Zhao et al. 2021; Qasim et al. 2021; Ma et al. 2022). Additionally, owing to the reliance on a single sales channel and traditional practices, continuous monocultivation has become prevalent, leading to soil degradation and an escalation of soilborne diseases in greenhouse vegetable fields (Daguerre et al. 2014; Ding et al. 2017).

Soil microorganisms are sensitive indicators of environmental change (Kumar et al. 2018). The soil pH significantly decreases, and the soil nutrient proportion becomes unbalanced with increasing cultivation years and nitrate N leaching, which ultimately leads to changes in the soil microbial community (Lv et al. 2019; Huang et al. 2019). Soil acidification increases the number of acidophilic fungi and root‐knot nematodes while decreasing the number of neutrophilic bacteria and the overall diversity of soil microorganisms. As a result, the resistance of the soil microbial community is reduced, allowing for the proliferation of soil‐borne pathogens (Van Elsas et al. 2012; Hu et al. 2023). In contrast, a healthy greenhouse vegetable production system is characterised by a diverse range of beneficial microorganisms that can produce various antibiotics to prevent fungal diseases (Kinsella and Schulthessm 2010; Qiu et al. 2012). However, most studies on continuous cropping obstacles have focused on the deterioration of soil quality and the proliferation of pathogenic fungi, neglecting the inhibitory effects of beneficial microorganisms such as *Bacillus* and *Paenibacillus* on soil‐borne diseases (Cao et al. 2017). Additionally, while microbial diversity is commonly used as an indicator of soil health, it is often insufficient for understanding the functions of the microbiome, such as its impact on crop yield, nutrient availability and effectiveness in the prevention and control of soil‐borne diseases (Jiao et al. 2022). In order to explore the potential interactions among microbial taxa, soil microbial co‐occurrence network analysis can provide insights into the relationships between the soil microbial community and the control of soil‐borne diseases on the basis of resource availability (Banerjee et al. 2019; Qiu et al. 2021).

In this study, 27 separate soil samples were collected from greenhouses used for continuous tomato cultivation for 5, 10 and 20 years respectively, as well as from the adjacent maize–wheat fields (MW). The soil microbial community diversity, network complexity and relative abundance of fungal genera associated with putative plant pathogens and bacterial genera associated with putative antagonism were analysed via high‐throughput sequencing to evaluate the effects of planting duration on soil health. The main objectives of this study were as follows: (1) to quantify changes in soil microbial diversity and network complexity under different years of cultivation shifting from croplands to greenhouses and (2) to evaluate the patterns of change in putative pathogenic fungi and putative antagonistic bacteria with respect to different planting years and to determine the role of soil putative antagonistic bacteria in the soil microbial community and their relationship with reducing the abundance of putative pathogenic fungi and the risk of soil‐borne diseases.

## 2 Materials and Methods

### 2.1 Description of the Study Area and Site Selection

The study area was situated in Shouguang County, Shandong Province (118°73′ E, 36°88′ N), renowned as the ‘hometown of sunken greenhouse vegetables in China’. The region experiences a typical continental monsoon climate, with an average annual temperature of approximately 12.4°C and annual precipitation of approximately 592 mm. The topsoil (0–30 cm) consisted of sandy loam, with sand (20–2000 μm), silt (2–20 μm) and clay (< 2 μm) accounting for 57%, 39% and 4% of the soil composition, respectively (Table 1).

**TABLE 1:** Soil properties at the 0–30 cm depth in tomato fields with varying years of cultivation and adjacent maize‐wheat fields (MW).

	Nmin	Olsen‐P	Avai. K	pH	EC	%		
	mg N kg^−1^	mg P kg^−1^	mg K kg^−1^		μS cm^−1^	Sand	Silt	Clay
MW	19 ± 1d	22 ± 1c	190 ± 9c	8.5 ± 0.03a	122 ± 6c	61 ± 1	35 ± 1	4 ± 0.1
5 year	48 ± 4c	103 ± 5b	303 ± 15b	8.3 ± 0.04a	416 ± 17a	58 ± 2	39 ± 2	4 ± 0.2
10 year	65 ± 5b	145 ± 9a	436 ± 16a	8.2 ± 0.04b	415 ± 29a	53 ± 2	42 ± 2	4 ± 0.2
20 year	83 ± 6a	162 ± 6a	456 ± 23a	7.5 ± 0.07b	358 ± 15b	56 ± 2	40 ± 2	4 ± 0.2

*Note:* Values are expressed as the means ± error (*n* = 27).

Based on 30 years of land‐use satellite imagery and consultation with local agronomists, nine greenhouses planted for 5, 10 or 20 years, together with nine adjacent maize–wheat fields, were selected across three neighbouring villages. The criteria for greenhouse selection were as follows: (1) The selected greenhouses were exclusively used for tomato cultivation, with two growing seasons per year—winter–spring and autumn–winter. (2) The methods employed for tomato planting, harvesting and field management, including irrigation and fertilisation practices, were generally consistent. (3) Within each village, three greenhouses were chosen, each with a different planting duration (5, 10 and 20 years), and these greenhouses were located within a radius of approximately 2–3 km, thus minimising the potential influence of soil and climate variations on the findings. We originally intended to collect samples from greenhouses newly established for precisely 1 year as control. Unfortunately, we were unable to secure enough qualifying greenhouses. Consequently, we substituted adjacent corn‐wheat field soil samples, which should be treated strictly as reference data rather than formal controls in this study. The maize–wheat plots were situated within 500 m of the corresponding greenhouses.

### 2.2 Field Management of Greenhouse Tomato and Maize–Wheat

During the winter–spring growing seasons, the tomato seedlings were transplanted in early January and harvested in mid‐June. A 6‐week fallow period followed during the summer due to high temperatures and heavy rainfall. To eliminate soil‐borne diseases, greenhouses are typically sealed for 3–4 weeks to conduct thermal disinfection (solarization), during which no crops are cultivated. For the autumn–winter growing seasons, tomato cultivation commenced in early August, with the harvest occurring in late December. The row spacing was 0.50 m, walkway width 0.60 m, and in‐row plant spacing 0.45 m. The locally common tomato variety *Qidali* was used in this experiment. One‐month‐old tomato seedlings obtained from Shouguang Jinhao Seed Industry Co. Ltd. were transplanted into the raised beds using a handheld transplanting tool in double rows. This cultivar is not grafted and is bright red with hard fruit and holds up well during storage and transportation. Traditional flood irrigation applied approximately 1400 mm of water per year, delivered in ~70 mm events. The total annual application rates for chemical fertilisers are 2000 kg N ha^−1^, 1400 kg P ha^−1^ and 1600 kg K ha^−1^. Compound fertiliser (ratio of N:P_2_O_5_:K_2_O is 16:15:16) and urea are the fertiliser types most frequently employed by local farmers. Furthermore, 800 kg N ha^−1^ organic fertiliser derived from chicken manure was applied prior to transplanting (Wan 2022).

The adjacent fields were planted with winter wheat (from mid‐October to the end of June) and summer maize (from July to the end of September). The average annual irrigation amount for winter wheat is approximately 300 mm. The annual nitrogen fertiliser application amount is approximately 500 kg N ha^−1^, with winter wheat and summer maize receiving equal shares of 50% each (Wan 2022).

### 2.3 Soil Sample Collection and Analysis

Following the tomato harvest in December 2019, we collected three independent samples of surface soil (0 ~ 30 cm) from each greenhouse. Within each greenhouse, sampling points were evenly distributed along the west–center–east transect; soils from south, centre and north positions were then composited to form one replicate. Each greenhouse was repeated three times (*n* = 3; Figure S1). Immediately after collection, samples were segregated and sealed in sterile bags, ensuring no exposure to the external environment, thereby preserving their pristine condition to the fullest extent possible. In December 2019, we concurrently implemented the same sampling procedure for the adjacent maize–wheat fields. The soil samples were then mixed separately from the north, middle and south directions, resulting in three independent soil samples for each direction. After being passed through a 2 mm sieve, the soil samples were divided into two parts and stored at −20°C and −80°C in a refrigerator. These samples were intended for the subsequent analysis of soil physicochemical properties and molecular biology.

To determine the nitrate and ammonium concentrations, a 0.01 m CaCl_2_ solution was used to extract fresh soil, and we used an AA3 autoanalyser (Luebe, Nordstadt Hamburg, Germany) for analysis. An ultraviolet spectrophotometer (UV1240, Shimadzu, Japan) at a wavelength of 880 nm was used to determine the soil available phosphorus concentration, whereas a flame photometer (Sherwood 410, England) was used for the determination of the soil available potassium concentration. The soil particle composition was determined via a laser particle size analyser (Mastersizer 2000, Malvern, England). The soil pH and EC were measured via a pH meter (IS126, Insmark, Shanghai) and conductivity meter (DDSJ‐308, Leizi, Shanghai), respectively, with a soil:water ratio of 1:5.

For soil DNA extraction, 0.5 g of each freeze‐dried sample was used in accordance with the FastDNA Kit (MP Biomedicals LLC, Santa Ana, CA) operating manual. The concentration and quality of the extracted DNA were measured via a NanoDrop ND‐2000c ultramicucleic acid protein analyser (NanoDrop Technologies, Wilmington, DE, USA). The qualified DNA samples were then stored at −80°C.

To amplify the V4 region of bacterial 16S rRNA, we used the primers barcoded‐515F (GTGCCAGCMGCCGCGGTAA) and barcoded‐806R (GGACTACVSGGGTATCTAAT) (Bergmann et al. 2011) for bacterial analysis. For fungal analysis, the primers barcoded‐ITS1 (CTTGGTCATTTAGAGGAAGTAA) and barcoded‐ITS2 (TGCGTTCTTCATCGATGC) (Buee et al. 2009; Zhuang et al. 2020) were used to amplify specific ITS target gene segments in the soil sample DNA. The PCR products were purified and sequenced via a PCR product purification kit from Beijing Aidlab Biotechnologies Co. Ltd., and the Illumina Nova6000 platform (Majorbio, Shanghai, China) was utilised for sequencing. For detailed information on the molecular biology experiments, refer to Hu et al. (2023).

### 2.4 Bioinformatics Analysis

Quality control of raw paired‐end reads was first conducted with fastp (version 0.14.1, https://github.com/OpenGene/fastp). Second, the primers were removed on the basis of primer information at the beginning and end of the sequences via Cutadapt software (https://github.com/marcelm/cutadapt/) to obtain paired‐end clean reads after quality control. Next, usearch‐fastq_mergepairs (V10, http://www.drive5.com/usearch/) was used to filter out the nonconforming tags and obtain the original splicing sequences (raw tags). Finally, OTU clustering with a 97% identification threshold was performed via UPARSE, and species annotation information was obtained by combining the SILVA (16S, version 138; Quast et al. 2013) and Unite (ITS, version 8.0; Kõljalg et al. 2013) databases to determine the taxonomic identity of all the sequence species. Given that this study primarily concentrates on analysing ecological patterns at higher taxonomic levels (e.g., abundance changes at the genus or phylum level), the OTU clustering results are sufficiently adequate for low‐resolution research requirements. Alpha diversity was characterised by the Shannon index and Chao1 index, whereas beta diversity was evaluated via the Bray–Curtis distance. The rarefaction was performed at a depth of 52,360 sequences (bacteria) and 14,884 sequences (fungi) per sample to normalise the sequencing effort across all samples. This threshold was chosen based on the 90% quantile of the minimum sequencing depth observed in the dataset, ensuring that > 95% of samples retained sufficient data after rarefaction. The construction and data analysis of networks were performed via the MENA (Molecular Ecological Network Analysis) platform (Deng et al. 2012; http://ieg4.rccc.ou.edu/mena). The MENA platform leverages RMT (Random Matrix Theory) to analyse correlation matrices (Spearman correlation matrices) constructed from microbial abundance data. By comparing the eigenvalue distribution of these matrices with that of random matrices, MENA automatically identifies statistically significant strong correlations (i.e., biological signals distinct from random noise), without requiring manually defined *p* value thresholds. This approach effectively circumvents the conservative bias introduced by traditional multiple hypothesis correction methods (e.g., BH or Bonferroni corrections), thereby addressing the challenge of high false positive rates in high‐dimensional datasets. The results were visualised via Cytoscape (version 3.8.2). The sequencing data can be accessed online (http://www.ncbi.nlm.nih.gov/), with accession numbers PRJNA1148087 for bacteria and PRJNA1148122 for fungi.

### 2.5 Statistical Analysis

Statistical analysis was performed via SAS 8.0 (SAS Institute Inc., USA). Shapiro–Wilk normality and Levene's test were used to check the normality and homogeneity of variance of all the data. The effects of different planting durations on the soil microbial community composition and the relative abundances of fungal genera associated with putative plant pathogens and bacterial genera associated with putative antagonism were analysed via one‐way ANOVA. The significance level was set at 0.05. OriginPro 2021 (OriginLab Corporation, Northampton, USA) was used for data visualisation. Pearson correlation analysis was conducted to assess the correlation between soil properties and soil microbial diversity using PROC CORR in SAS. Structural equation model analysis was performed via AMOS 22.0 software (AMOS Development Corporation, Chicago, USA). The data underwent preprocessing to systematically evaluate and address missing values, outliers and normality assumptions. The model parameters were estimated using the maximum likelihood estimation method, while ensuring that the fit indices fell within acceptable limits. Alpha diversity (Shannon index and Chao1 index), principal coordinate analysis (PCoA) and network analysis were performed on the basis of the OTU abundance table. The one‐way ANOVA was used to analyse the variance of treatments for microbial community structure and Shannon index using R software (R Core Team 2024, version 4.1.0). The PCoA analysis was performed using the Bray–Curtis distance algorithm to compute the sample distance matrix, and the ANOSIM test method was employed to assess the significance of changes in community structure (*p* = 0.001). The vegan R package (v.2.5–2) was used for data processing and statistical analysis (Oksanen et al. 2013).

## 3 Results

### 3.1 Soil Chemical Properties, Microbial Diversity and Community Composition

Although there was a certain spatial distance (< 2 km) between the sampling sites, there was minimal difference in the contents of sand, silt and clay (Table 1 and Figure S2). However, the contents of soil Nmin, Olsen‐P and available potassium significantly increased with the prolonged duration of conversion from croplands to greenhouse cultivation, and these values were significantly greater than those in adjacent maize–wheat fields. The soil pH decreased significantly with increasing years of tomato cultivation (Table 1 and Figure S2).

**FIGURE 1:**
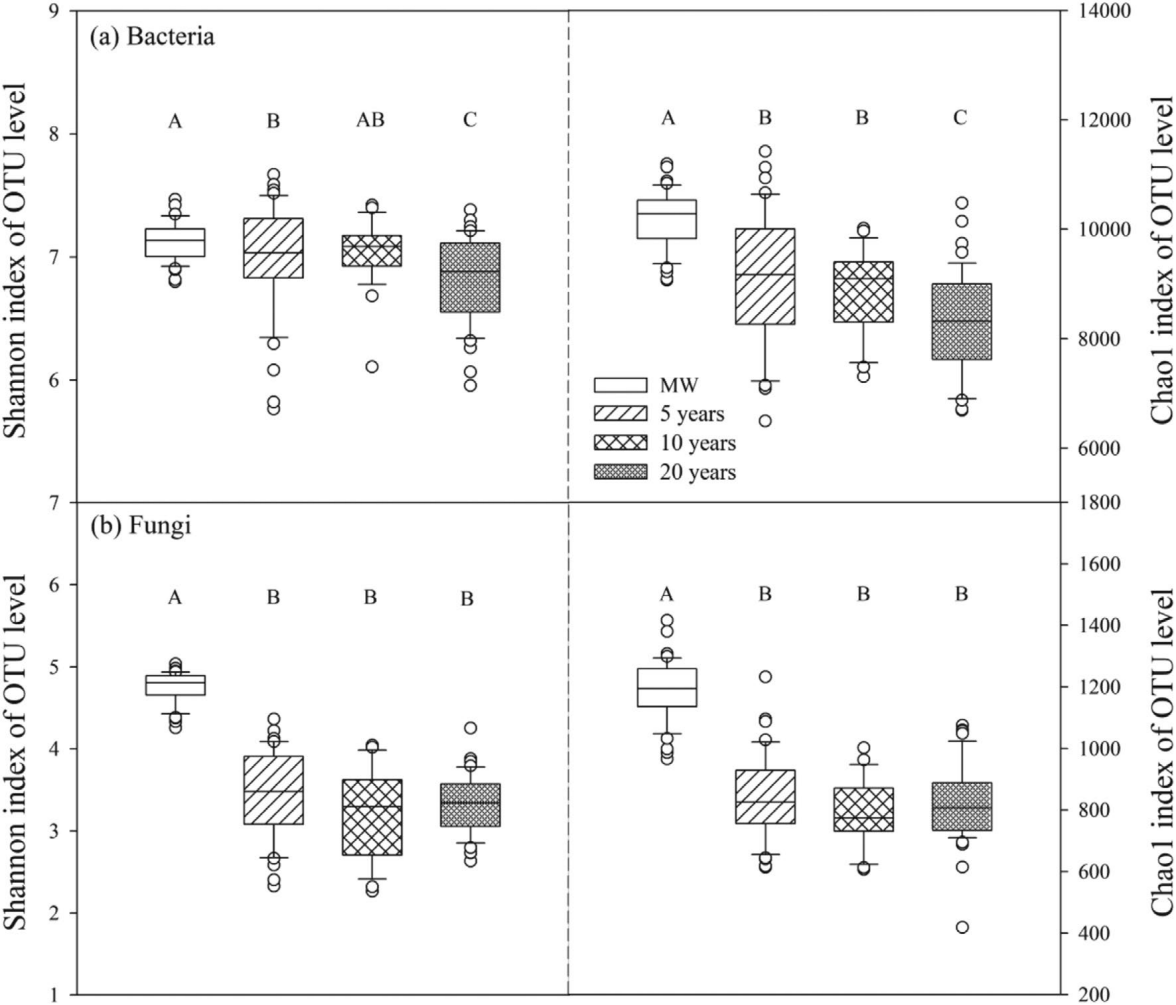
Shannon and Chao1 indices of soil bacteria (a) and fungi (b) at the OTU level under different years of cultivation transitioning from croplands to greenhouses (*n* = 27). Different capital letters represent significant differences (*p* < 0.05) among cultivation years. The boundaries of the boxes indicate the first and third quartiles; the lines and squares within the boxes represent the medians. Whiskers mark the 10% and 90% percentiles, and the outliers are shown as dots. The error bars represent the standard error of the mean.

**FIGURE 2:**
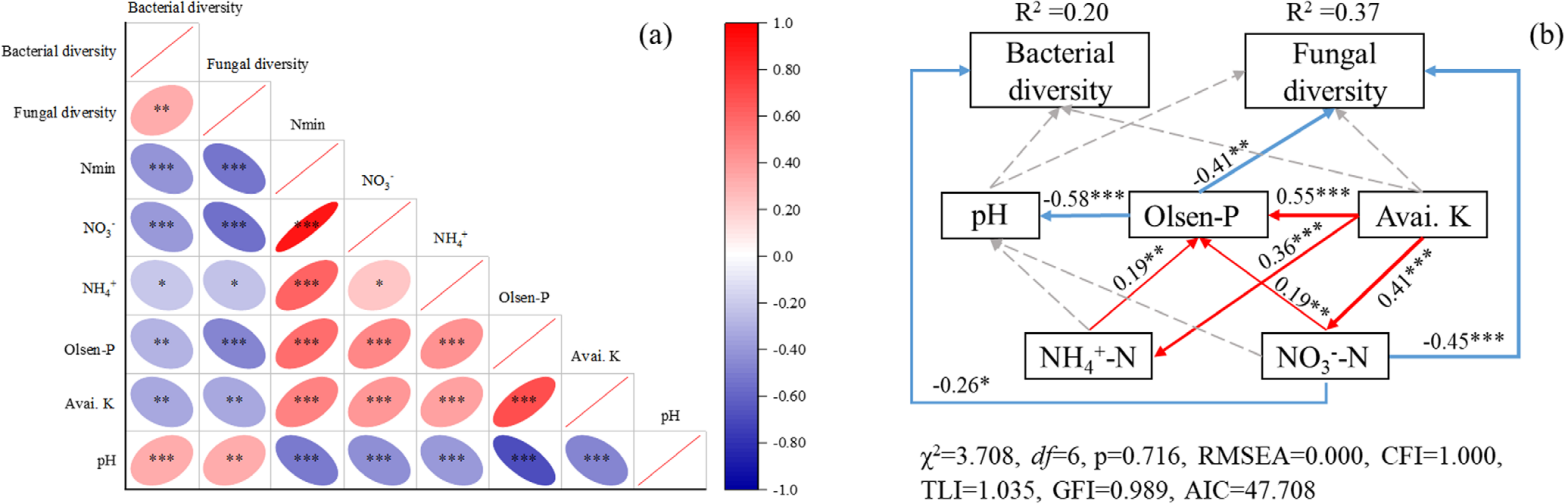
The relationships between microbial diversity and soil parameters (a), as well as the structural equation model depicting the response of microbial diversity to the concentration of soil substrates, including Olsen‐P, available K, nitrification (NH_4_^+^), denitrification (NO_3_^−^) and soil pH (b). Red indicates a positive correlation, whereas blue indicates a negative correlation. *, ** and *** indicate significant effects at *p* = 0.05, 0.01 and 0.001, respectively.

Upon conducting statistical analysis of the sequencing data, it was determined that the total number of sequences following bacterial community optimisation amounted to 21,738,429, with an average sequence length of 291 bp. Additionally, the total number of operational taxonomic units (OTUs) was 15,571. The total number of sequences after quality check of the fungal community was 17,179,816, with an average sequence length of 288 bp and a total number of OTUs of 6673. The Shannon index and Chao1 index of soil bacteria and fungi in the tomato field at the OTU level were significantly lower than those in the maize–wheat field (MW) (Figure 1). With the increasing duration of conversion from croplands to greenhouses cultivation, the Shannon index and Chao1 index of bacteria significantly decreased (Figure 1a). However, there was no significant difference in the Shannon index or Chao1 index of fungi among the planting years (Figure 1b). The Shannon indices of bacteria and fungi were significantly positively correlated with soil pH (*p* < 0.001 and *p* = 0.001) but negatively correlated with soil Nmin (*p* < 0.001), Olsen‐P (*p* = 0.003 and *p* < 0.001) and Avai. K (*p* = 0.001) contents (Figure 2a). According to the results of structural equation modelling, the path coefficients of the soil NO_3_^−^‐N content with the bacterial and fungal diversity indices were −0.26 and −0.45, respectively, and the path coefficients of the soil phosphorus content and fungal diversity index were −0.41 (*p* = 0.716, not significant) (Figure 2b).

**FIGURE 3:**
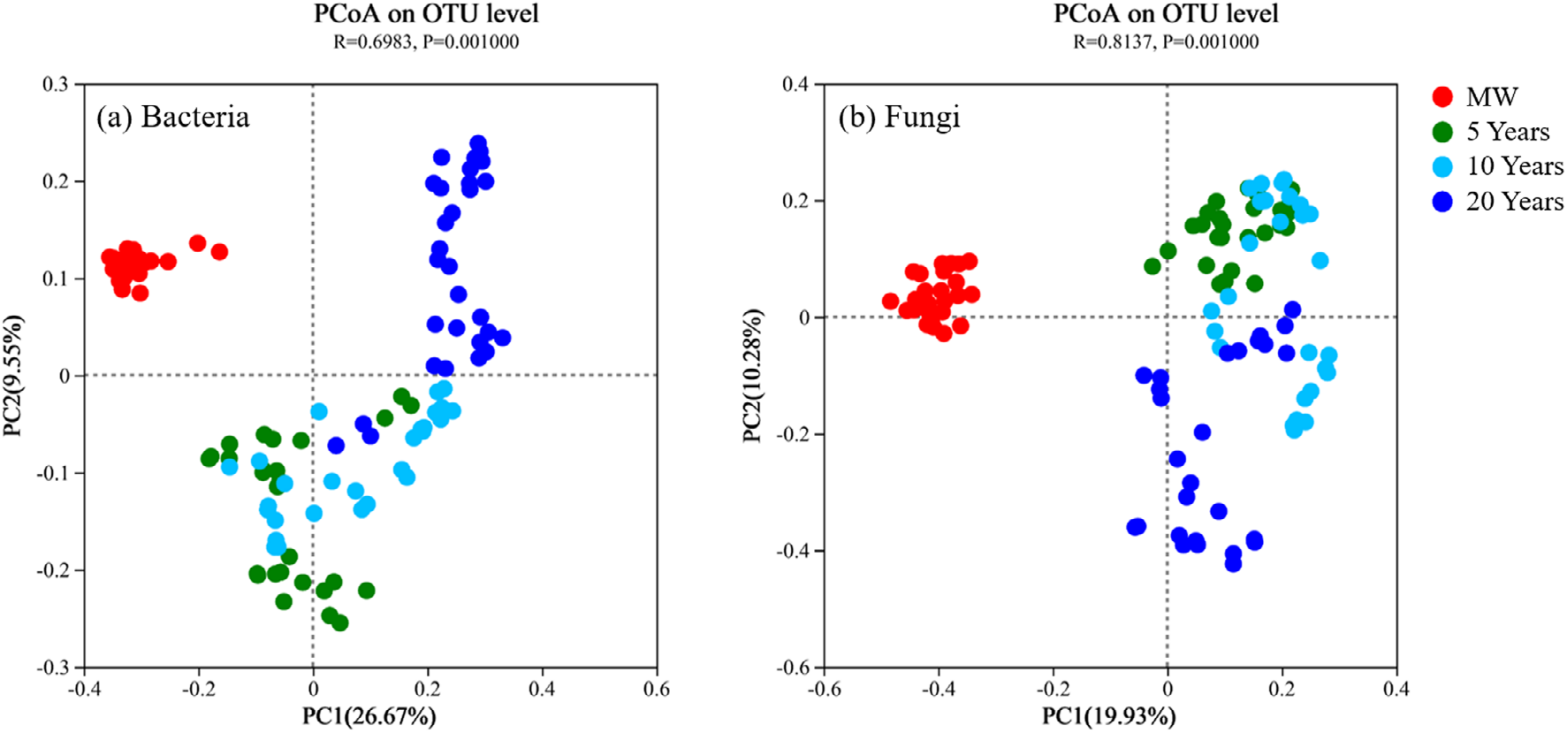
Principal coordinate analysis of the bacterial (a) and fungal (b) communities under different planting durations at the OTU level (*n* = 27).

**FIGURE 4:**
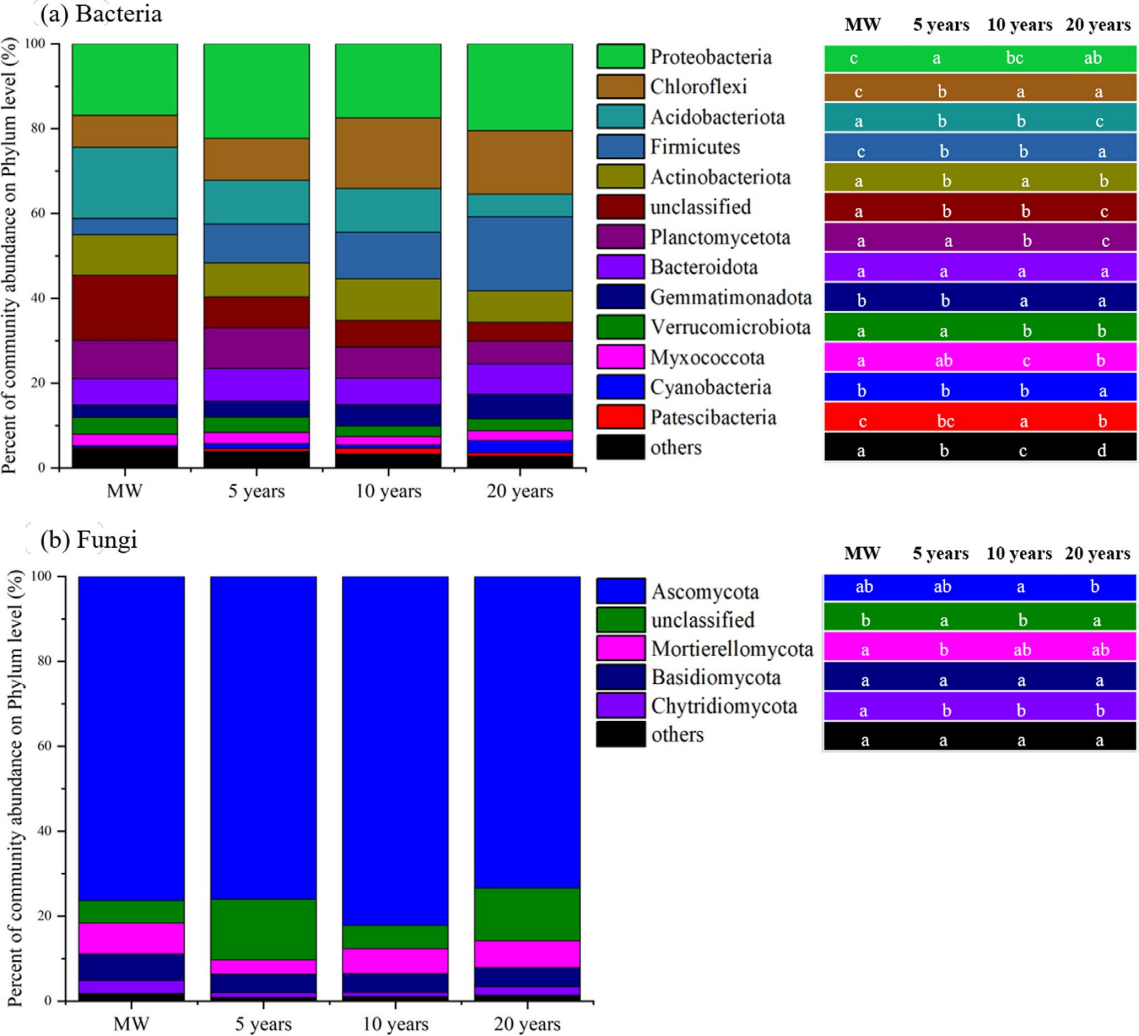
Soil bacterial (a) and fungal (b) community bar plot analysis under different planting durations at the phylum level (*n* = 27). Others = the relative abundance was ≤ 1%. The different lowercase letters represent significant differences (*p* < 0.05) among the different planting years.

Principal coordinate analysis (PCoA) revealed that the composition of the soil bacterial and fungal communities significantly clustered across the different planting years (Figure 3). Axis 1 and axis 2 explained 26.7% and 9.6% of the total variation in the bacterial communities, respectively (Figure 3a; *R* = 0.6983, *p* = 0.001), and 19.9% and 10.3%, respectively, of the fungal communities (Figure 3b; *R* = 0.8137, *p* = 0.001). Pairwise ANOSIM revealed significant differences in both bacterial (Figure S3; *p* = 0.001) and fungal (Figure S4; *p* = 0.001) composition between any two planting years. In the bacterial community, the top 9 bacteria in terms of relative abundance at the phylum level accounted for approximately 90% of all identified phyla (Figure 4a). With the increasing duration of conversion from croplands to greenhouse cultivation, the relative abundances of Acidobacteria (*p* < 0.001), Actinobacteria (*p* = 0.001), and Planctomycete (*p* < 0.001) significantly decreased, whereas the relative abundances of Proteobacteria (*p* = 0.001), Chloroflexi (*p* < 0.001), Firmicutes (*p* < 0.001) and Gemmatimonadetes (*p* < 0.001) increased significantly (Figure 4a). In the fungal communities, the relative abundances of Ascomycota, Mortierellomycota and undefined fungi accounted for approximately 90% of all identified phyla (Figure 4b). The relative abundance of Mortierellomycota significantly decreased after 5 years of continuous cropping (Figure 4b; *p* = 0.032). The relative abundance at the genus level was presented in Figure S5. With the increasing duration of conversion from croplands to greenhouse cultivation, the relative abundances of *Bacillus* (*p* < 0.001), *Pirellula* (*p* < 0.001), *Sphingomonas* (*p* < 0.001), *Streptomyces* (*p* = 0.004) and *Paenibacillus* (*p* < 0.001) significantly decreased, whereas the relative abundances of *Aquicella* (*p* < 0.001) and *Nitrolancea* (*p* < 0.001) increased significantly (Table S1 and Figure S5a). Within the fungal community, the relative abundance of *Aspergillus*, *Mycothermus*, *Cephaliophora* and *Cladosporium* increased significantly after 5 years of continuous cropping, whereas the relative abundances of *Schizothecium, Pyrenochaetopsis*, among others, decreased significantly (Table S1 and Figure S5b; *p* < 0.001). We identified fungal genera associated with putative plant pathogens (Santhanam et al. 2015; Zhao et al. 2016) and bacterial genera associated with putative antagonism (Antonopoulos et al. 2008; Kinsella and Schulthessm 2010; Qiu et al. 2012) with a relative abundance exceeding 1% and relevance to tomatoes, and performed a detailed analysis of their abundance variations.

### 3.2 Soil Bacterial and Fungal Network Complexity

**FIGURE 5:**
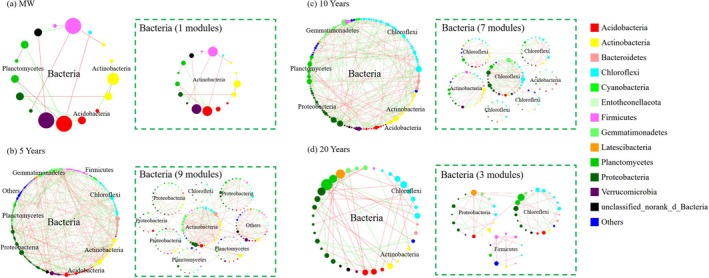
Co‐occurrence patterns of the bacterial community under different planting durations (*n* = 27). The co‐occurrence pattern of each subnetwork under different treatments. Subnetworks were generated for each treatment from the network by preserving the nodes and edges presented (nodes ≥ 10). The red and green lines represent positive and negative correlations, respectively. The data were fitted via linear regression and assessed via Spearman's rank correlation *ρ*.

Soil bacterial co‐occurrence patterns differed markedly among planting years (Figure 5 and Table S2). Relative to maize–wheat soil, greenhouse soil displayed a more complex network, with significantly more nodes, edges, large modules (≥ 10 nodes) and a higher mean degree. The complexity of the bacterial network was greatest after 5 years of continuous vegetable cultivation (Figure 5). The numbers of nodes, edges, and modules were 297, 394 and 9, respectively, in the 5‐year‐old soil, which were significantly greater than the 156, 142 and 3, respectively, in the 20‐year‐old soil (Figure 5 and Table S2). The complexity of the bacterial network was somewhere in between when it was cultivated continuously for 10 years.

**FIGURE 6:**
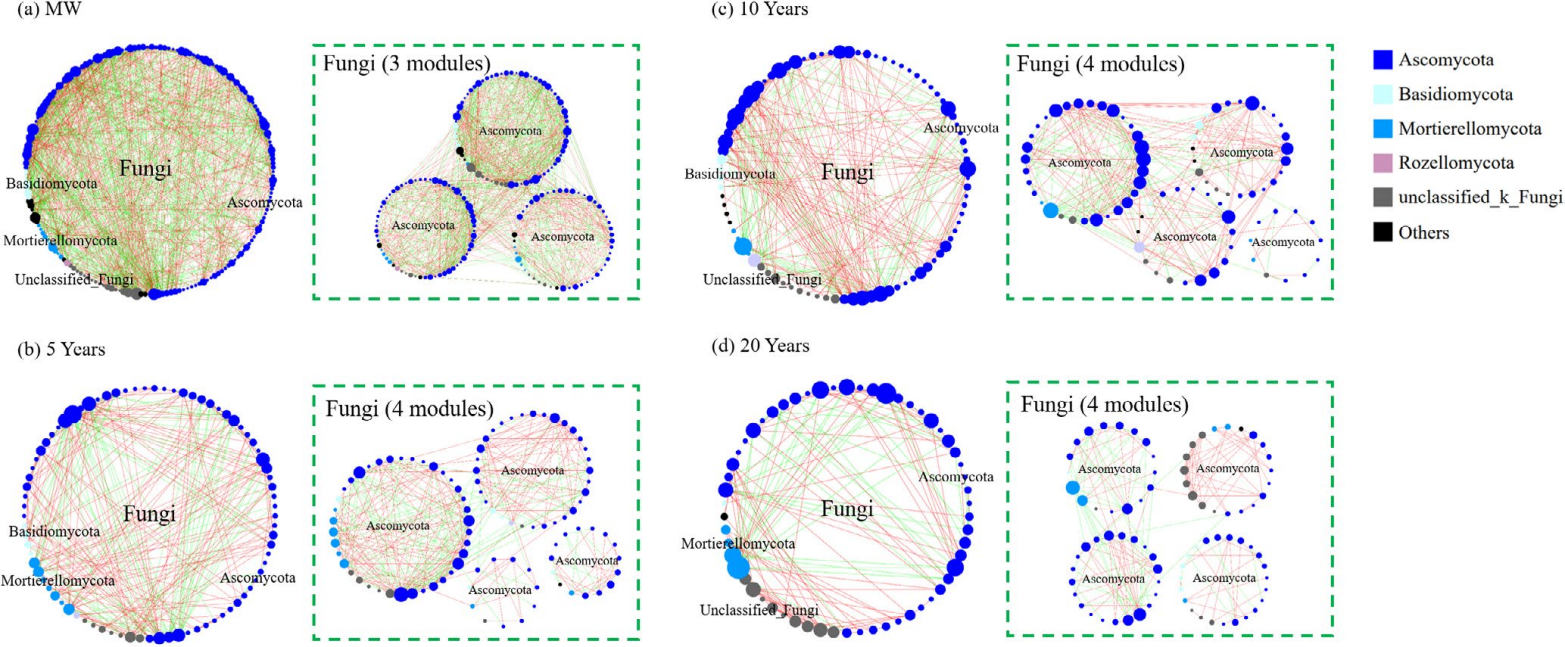
Co‐occurrence patterns of the fungal community under different years of cultivation (*n* = 27). The co‐occurrence pattern of each subnetwork under different treatments. Subnetworks were generated for each treatment from the network by preserving the nodes and edges presented (nodes ≥ 10). The red and green lines represent positive and negative correlations, respectively. The data were fitted via linear regression and assessed via Spearman's rank correlation *ρ*.

The co‐occurrence patterns of the fungal networks exhibited substantial variations across the different years of cultivation, as illustrated in Figure 6. The highest complexity of the fungal network was observed in the maize–wheat soil, with 220 nodes, 1215 edges and 3 modules (Figure 6; Table S3). These numbers decreased to 114, 343 and 4, respectively, in the 5‐year‐old soil and further decreased to 102, 303 and 4, respectively, in the 10‐year‐old soil. Notably, the complexity of the fungal network reached its lowest point in the 20‐year‐old soil, with 96 nodes, 211 edges and 4 modules (Figure 6 and Table S3).

### 3.3 Plant Pathogens and Antagonistic Bacteria

**FIGURE 7:**
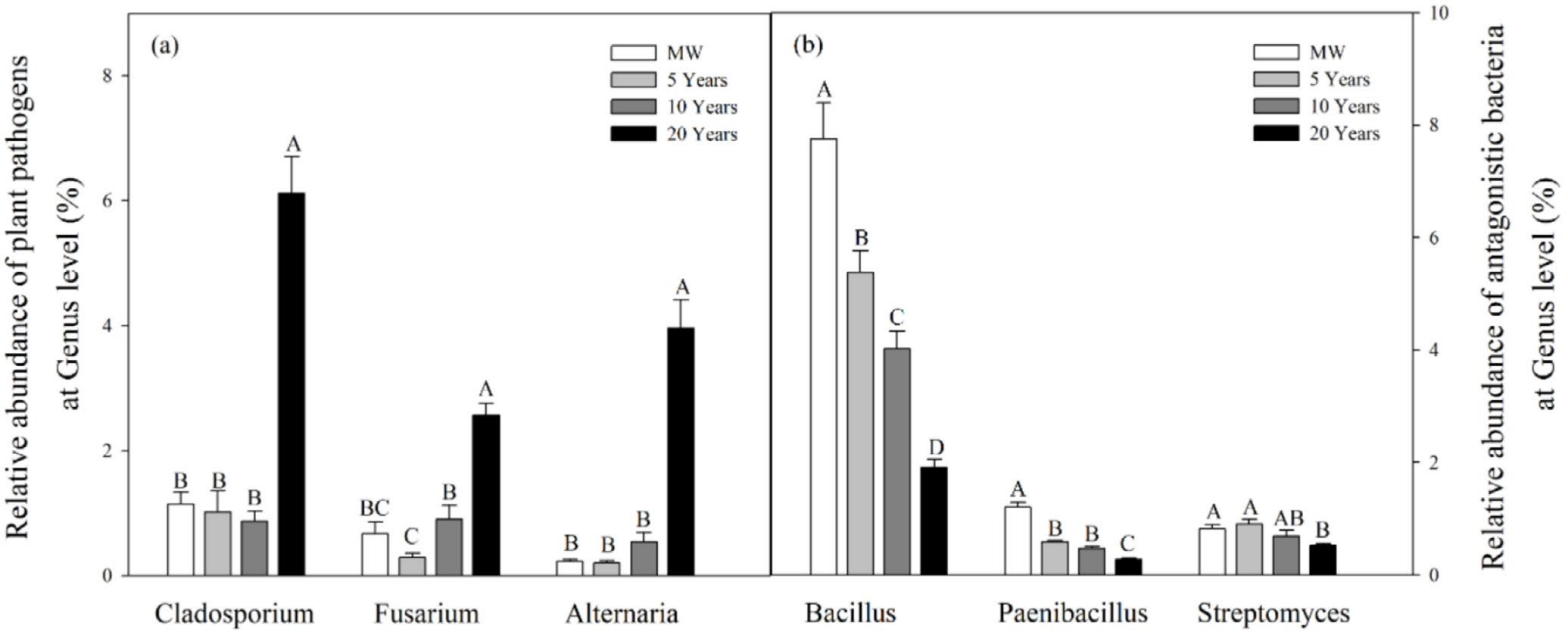
Relative abundance of putative plant pathogens (a) and putative antagonistic bacteria (b) at the genus level under different cultivation durations (*n* = 27). The different capital letters represent significant differences (*p* < 0.05) among the different cultivation years.

Relative to maize–wheat soil, the mean abundance of the putative leaf‐mould pathogen *Cladosporium* rose from 1.1% to 6.1% after 20 years of continuous tomato cultivation (Figure 7a, *p* = 0.001). *Fusarium*, a putative pathogen that causes tomato wilt disease, also significantly increased from 0.7% to 2.6%. Furthermore, the average relative abundance of the soil putative pathogen *Alternaria* significantly increased from 0.2% to 4.0%. No significant difference was observed in the number of putative pathogens mentioned above when comparing maize–wheat soil with vegetable soils after 5 and 10 years (Figure 7a, *p* = 0.361).

*Bacillus*, *Paenibacillus* and *Streptomyces* are bacterial genera associated with putative antagonism that can effectively suppress common soil‐borne diseases in tomatoes. However, the average relative abundance of these three putative antagonistic bacteria significantly decreased with increasing planting duration (Figure 7b, *p* < 0.001). Among them, *Bacillus* and *Paenibacillus* demonstrated the most substantial decreases over the 20‐year period, decreasing from 7.8% to 1.9% and from 1.2% to 0.3%, respectively.

## 4 Discussion

Excessive water and fertiliser inputs, as well as long‐term monoculture, are detrimental factors that lead to soil‐borne diseases, posing a serious threat to the sustainability of greenhouse vegetable production (Xiong et al. 2015; Lv et al. 2019). The potential link between decreases in soil microbial diversity and stability and the occurrence of soil‐borne diseases has always been a prominent area of research (Xiong and Lu 2022; Hu et al. 2023). Our findings indicate a significant reduction in the diversity of soil bacteria and fungi, as well as network complexity, along with a decrease in the relative abundance of putative beneficial microorganisms with prolonged cultivation shifting from croplands to greenhouses. These factors contribute to the proliferation of putative plant pathogens and the exacerbation of soil‐borne diseases (Daguerre et al. 2014; Huang et al. 2019).

### 4.1 Long‐Term Monoculture Reduced Soil Microbial Diversity and Network Complexity

A lower Shannon index and Chao1 index typically imply a lower species diversity within an ecosystem or community. Long‐term monoculture had a significant negative effect on the soil microbial diversity and richness indices, as well as the composition of the bacterial and fungal communities (Figures 1 and 3). This finding aligns with previous research indicating that as the number of years of cultivation increases, there is an accumulation of soil nutrients (table 1 and Figure S2; Lv et al. 2019; Wan 2022), resulting in the enrichment of specific microbial populations (e.g., eosinophilic fungi) and a decline in overall microbial diversity (Figure 1; Zhou et al. 2014; Huang et al. 2019). This correlation is supported by the negative relationship observed between the Shannon indices of bacteria and fungi and the soil nutrient content (Figure 2a). Structural equation modelling further revealed that the increase in soil NO_3_^−^‐N and Olsen‐P contents was the primary cause of the decline in microbial diversity (Figure 2b). Moreover, the excessive use of nitrogen fertilisers and irrigation has led to nitrate leaching, causing a significant decrease in soil pH (Lv et al. 2019). This low pH promotes the growth of acidophilic fungi and inhibits the growth of neutrophilic bacteria, resulting in further reduced microbial diversity (Figures 1 and 3; Hu et al. 2023). These factors may contribute to the decreased abundance of beneficial populations and the prevalence of soil‐borne diseases in greenhouse vegetable fields (Figures 4 and 7; Banerjee et al. 2020). It is noteworthy that, in contrast to bacterial communities, fungal diversity exhibited no significant variation across consecutive cultivation years (*p* = 0.285). This stability may be attributed to two key factors. First, the physical barrier function of fungal mycelia alleviates direct effects of environmental stressors (de Vries et al. 2018). Second, high functional redundancy of fungi ensures that shifts in dominant groups (e.g., the transition from saprophytic fungi to pathogenic fungi) do not alter diversity indices (de Menezes et al. 2017). This observation implies that addressing short‐term consecutive cropping challenges through micro‐ecological management should prioritise bacterial community imbalances. Nevertheless, long‐term monitoring remains essential for detecting potential structural shifts in fungal communities, such as the enrichment of *Fusarium* species. Although microbial diversity and community composition are important indicators of soil health, a more comprehensive understanding of the interactions between microorganisms and their potential associations with soil‐borne diseases can be gained through evaluating the complexity of soil microbial networks and the presence of keystone species (Qiu et al. 2021).

In the majority of cases, the size of nodes within microbial networks exhibits a positive correlation with their connection numbers (i.e., degree centrality), suggesting their potential to serve as keystone species in ecological functions (Banerjee et al. 2018). Modular analysis further enables the identification and clarification of key species within microbial networks at the OTU level (Figure S6). With increasing cultivation duration, there was an initial increase followed by a decrease in the complexity of the bacterial network and the number of keystone species. The peak was reached at 5 years of consecutive cultivation (Figures 5 and S6; Table S2). Compared to the prior findings in vineyards (Liu et al. 2021), the bacterial network complexity in the maize–wheat fields exhibited the lowest value in this experiment. We hypothesise that this may be attributed to the fact that the maize–wheat fields are not fallow land, where reduced soil disturbance would promote microbial interactions (Wang et al. 2024). Furthermore, a sunken solar greenhouse field has a deficiency of soil organic matter, which leads to low soil fertility (Wang et al. 2021). To meet the high demand for water and fertilisers for greenhouse vegetable growth, significant amounts of fertilisers and chicken manure have been applied (Wan et al. 2023), stimulating bacterial growth and interactions. As a result, the complexity of the bacterial network increased, and the number of keystone species also increased (Figure 5; Table S2; Herren and Mcmahon 2017). However, prolonged cultivation for more than 10 years results in excessive accumulation of soil nutrients (Table 1 and Figure S2). This not only weakens the interactions between microorganisms (Figure 5; Table S2; Ren et al. 2018; Bei et al. 2018; Wang et al. 2022) but also exacerbates soil acidification (Lv et al. 2019) and imposes physiological stress on soil microorganisms. Consequently, the number of keystone species and the stability of bacterial networks are reduced (Banerjee et al. 2019; Li, Roley, et al. 2021). In contrast to those of bacteria, the complexity of fungal networks and the number of key species decreased with increasing cultivation duration (Figure 6). This finding aligns with previous vineyard research indicating that fungal network complexity decreases with increasing cultivation years (Liu et al. 2021). The fungal network was more strongly correlated with the soil nutrient content and the C:N ratio (Bahram et al. 2018), as evidenced by the greater path coefficient between fungal diversity and the soil nitrate nitrogen and available phosphorus contents (Figure 2b). The soil nutrient content in maize–wheat fields is depleted (Table 1 and Figure S2), and fungi must compete for resources (de Menezes et al. 2017), resulting in a tighter and more complex fungal interaction network (Figure 6). Furthermore, prolonged cultivation in greenhouse vegetable fields leads to the overaccumulation of soil nutrients and a low C:N ratio, resulting in a significant reduction in the complexity of the fungal network (Tables 1 and S3; Figures S2 and 6). This increases susceptibility to pathogen invasion (Wei et al. 2015; Xiong and Lu 2022).

### 4.2 Microbial Network Complexity Is Related to the Relative Abundances of Plant Pathogenic Fungi and Antagonistic Bacteria

*Cladosporium*, *Fusarium* and *Alternaria* are common fungal genera associated with putative plant pathogens that cause soil‐borne diseases in tomatoes (Santhanam et al. 2015; Zhao et al. 2016). In greenhouse vegetable fields, the abundance of these three putative pathogenic fungi increased significantly, by six to tenfold, compared with that in maize–wheat fields after two decades of continuous cultivation (Figure 7a). This study revealed significant enrichment of *Fusarium* (0.3%–2.6%) and *Alternaria* (0.2%–4.0%) in soil under consecutive tomato cultivation. These ranges align with prior reports (*Fusarium*: 0.5%–8.2%; *Alternaria*: 0.1%–3.5%) (Jiménez‐Fernández et al. 2010; Wei et al. 2015). Notably, in plots exceeding 3 years of continuous cultivation, *Fusarium* relative abundance increased to > 3‐fold initial values (Ding et al. 2024). Clearly, the practice of rotating diverse crop varieties can effectively alleviate the adverse effects of pathogens on crop production by disrupting the interaction between plant hosts and pathogens (Zhou et al. 2023). The high‐temperature and high‐humidity conditions in greenhouses are critical factors contributing to the occurrence of soil‐borne diseases. For example, in Solanaceae cropping systems under continuous cultivation, the infection rate of *Phytophthora infestans* significantly increases when relative humidity exceeds 80% (Wang et al. 2018). Additionally, under these conditions, the decomposition rate of soil organic matter accelerates, leading to increased oxygen consumption during the decomposition process (Li, Roley, et al. 2021; Li, Yang, et al. 2021). This can exacerbate hypoxic conditions in greenhouse soils, thereby promoting the anaerobic metabolic pathways of putative pathogenic microorganisms, such as *Fusarium* species, and ultimately disrupting the balance of soil microecology (Huang et al. 2019). Besides, continuous cultivation can be attributed to soil acidification, imbalanced mineral nutrient levels and excessive water and fertiliser application (Table 1 and Figure S2). These factors not only decrease microbial diversity but also shift the soil microbial community from being predominantly ‘bacterial type’ to ‘fungal type’ (Berendsen et al. 2012). As a result, fungal genera associated with putative plant pathogens thrive (Figure 7a). Furthermore, the reduction in soil microbial network complexity and the abundance of keystone species weaken the interactions and suppressive abilities of microorganisms, leading to a significant increase in the relative abundance of putative fungal pathogens (Figures 5, 6 and 7a; Hernandez et al. 2021).

On the other hand, bacteria synthesise a diverse range of antifungal compounds via secondary metabolism, including antibiotics, volatile organic compounds and siderophores (Crits‐Christoph et al. 2018). These compounds directly suppress fungal growth or disrupt their cellular structures, thereby enhancing resistance against soil‐borne pathogens and contributing to the stabilisation of soil microecology (Jiao et al. 2022). For instance, *Bacillus* species produce antibiotics such as *iturin A* that effectively suppress soil‐borne fungal diseases, including *Fusarium wilt* (Kinsella and Schulthessm 2010). *Paenibacillus* synthesises *polymyxin* and other antibiotics inhibiting the formation of microsclerotia in *Verticillium dahliae*—a key survival structure of *wilt* disease (Antonopoulos et al. 2008). *Streptomyces* generates metabolites including *tubercidin* and *candicidin*, which demonstrate significant inhibitory effects against both *Fusarium wilt* and *Cladosporium leaf mould* (Qiu et al. 2012). However, soil acidification and secondary salinisation hinder microbial enzyme activity and the growth of bacterial genera associated with putative antagonism such as *Bacillus*, *Paenibacillus* and *Streptomyces* (Xiong et al. 2015). Consequently, the relative abundance of these beneficial bacteria decreased with increasing duration (Figure 7b). This finding aligns with the reported patterns under inorganic fertilisation (Li et al. 2024). In contrast, organic management increases the abundances of these three genera under continuous cultivation (Luo et al. 2023). As a result, the availability of secondary metabolites that effectively prevent fungal diseases, which pose a serious threat to sustainable vegetable production, is significantly reduced (Qiu et al. 2012). Due to the inherent constraints of the sequencing methodology utilised in this study, a comprehensive evaluation of the relationships between microbial gene expression, enzyme activity, metabolite profiles and the potential for soil‐borne disease suppression could not be fully achieved. Furthermore, this study focused on analysing the dynamics of putative plant‐pathogenic and antagonistic microorganisms using microbial community composition data. However, the absence of direct field evidence regarding disease occurrence (e.g., incidence or severity metrics) represents a significant limitation. Future research should focus on further investigating these factors and experimentally validating their specific contributions to disease management.

## 5 Conclusion

This study demonstrates that converting croplands to greenhouse cultivation reduces soil microbial diversity, network complexity and the abundance of beneficial microorganisms. These findings further reveal a significant increase in the relative abundance of putatively plant pathogenic fungal genera, contributing to frequent soil‐borne diseases. To ensure sustainable greenhouse vegetable production, addressing these challenges is crucial. Measures such as reducing water and fertiliser overuse, preventing soil acidification and nutrient imbalance and maintaining an appropriate soil C:N ratio are essential to establish a healthy soil microbial community and increase plant resistance. These insights provide putative pathways to overcome technical barriers in intensive agriculture.

## Author Contributions

**Jing Hu:** funding acquisition, writing – original draft, writing – review and editing. **Li Wan:** investigation. **Yafang Wang:** investigation. **Kuai Dai:** writing – review and editing. **Dr. Klaus Butterbach‐Bahl:** writing – review and editing. **Shan Lin:** writing – review and editing, funding acquisition.

## Conflicts of Interest

The authors declare no conflicts of interest.

## Supporting information


**Data S1:** emi470165‐sup‐0001‐Supinfo.

## Data Availability

The data that support the findings of this study are available on request from the corresponding author. The data are not publicly available due to privacy or ethical restrictions.
