# Supplementary material for: The Decrease of Soil Microbial Community Diversity and Network Complexity Results in the Increase of Soil‐Borne Diseases With Monocultural Years in Greenhouse Tomato Production Systems
Source: Environ Microbiol Rep. 2025 Jul 24;17(4):e70165. doi: 10.1111/1758-2229.70165 (PMC12287619; doi:10.1111/1758-2229.70165)
Supplement: Supplementary file 1 — Data S1: emi470165‐sup‐0001‐Supinfo. [file EMI4-17-e70165-s001.docx]

**Supplementary**

Table S1 Relative abundance of soil bacteria and fungi at the genus level in tomato fields with varying years of cultivation, and adjacent maize-wheat fields (MW).

|  | **Genus** | **MW** | **5 Years** | **10 Years** | **20 Years** | ***p*-value** |
| --- | --- | --- | --- | --- | --- | --- |
| Bacteria | Bacillus | 7.76a | 5.38b | 4.02c | 1.91d | < 0.001 |
|  | RB41 | 3.06a | 1.75b | 2.55a | 0.91c | < 0.001 |
|  | Pseudomonas | 0.50c | 2.34a | 1.05bc | 1.75ab | 0.006 |
|  | Steroidobacter | 0.57c | 1.40a | 1.19a | 0.89b | < 0.001 |
|  | Pirellula | 1.03a | 0.98a | 0.72b | 0.59b | < 0.001 |
|  | Sphingomonas | 1.03a | 0.91a | 0.83a | 0.51b | < 0.001 |
|  | Pir4_lineage | 0.83b | 1.04a | 0.74b | 0.39c | < 0.001 |
|  | Streptomyces | 0.82a | 0.91a | 0.69ab | 0.52b | 0.004 |
|  | Subgroup_10 | 0.42b | 1.00a | 0.90a | 0.36b | < 0.001 |
|  | Paenibacillus | 1.20a | 0.59b | 0.47b | 0.28c | < 0.001 |
|  | Aquicella | 0.20b | 0.30b | 0.4b | 1.33a | < 0.001 |
|  | Nitrolancea | 0.11c | 0.15c | 0.42b | 1.00a | < 0.001 |
| Fungi | Aspergillus | 1.20b | 1.98b | 4.82b | 15.58a | < 0.001 |
|  | Lophotrichus | 1.73b | 7.00a | 6.16a | 7.03a | < 0.001 |
|  | Mortierella | 6.92a | 3.29b | 5.17ab | 6.06ab | 0.083 |
|  | Mycothermus | 1.49b | 1.93b | 2.95b | 13.80a | < 0.001 |
|  | Cephaliophora | 0.65c | 0.99c | 4.79b | 8.56a | < 0.001 |
|  | Conocybe | 0.70a | 2.99a | 2.08a | 3.49a | 0.385 |
|  | Cladosporium | 1.14b | 1.02b | 0.87b | 6.12a | < 0.001 |
|  | Ascobolus | 0.50c | 4.48a | 2.31b | 0.69c | < 0.001 |
|  | Chaetomium | 1.69bc | 3.21a | 1.95b | 0.65c | < 0.001 |
|  | Schizothecium | 4.98a | 0.35b | 0.35b | 1.14b | < 0.001 |
|  | Arachniotus | 0.54b | 0.47b | 1.38b | 3.96a | < 0.001 |
|  | Acremonium | 1.36ab | 0.78b | 0.97b | 2.83a | 0.068 |
|  | Emericellopsis | 0.47b | 0.66b | 0.31b | 4.04a | < 0.001 |
|  | Myceliophthora | 0.30b | 1.61ab | 2.26a | 1.18ab | 0.020 |
|  | Alternaria | 0.23b | 0.20b | 0.54b | 3.96a | < 0.001 |
|  | Fusarium | 0.67bc | 0.29c | 0.90b | 2.57a | < 0.001 |
|  | Pyrenochaetopsis | 3.83a | 0.21b | 0.13b | 0.19b | < 0.001 |
|  | Chrysosporium | 0.16b | 1.04a | 0.65ab | 0.10b | 0.065 |
|  | Zopfiella | 0.40b | 0.22b | 0.17b | 1.00a | 0.022 |
|  | Sarocladium | 1.35a | 0.13b | 0.09b | 0.10b | < 0.001 |
|  | Podospora | 1.40a | 0.11b | 0.05b | 0.06b | < 0.001 |
|  | Solicoccozyma | 1.26a | 0.08b | 0.05b | 0.07b | < 0.001 |
|  | Gibberella | 1.15a | 0.08b | 0.04b | 0.09b | < 0.001 |
|  | Talaromyces | 1.09a | 0.10b | 0.06b | 0.09b | < 0.001 |

Note: Values are expressed as the means (n=27).

Table S2 The characteristics of empirical bacterial network structure constructed under different planting years

| **Network Indexes** | **MW** | **5 Years** | **10 Years** | **20 Years** |
| --- | --- | --- | --- | --- |
| Total nodes | 128 | 297 | 221 | 156 |
| Total links | 86 | 394 | 269 | 142 |
| R square of power-law | 0.812 | 0.926 | 0.928 | 0.947 |
| Average degree (avgK) | 1.344 | 2.653 | 2.434 | 1.821 |
| Average clustering coefficient (avgCC) | 0.039 | 0.089 | 0.126 | 0.087 |
| Average path distance (GD) | 2.985 | 5.601 | 5.758 | 4.702 |
| Geodesic efficiency (E) | 0.529 | 0.221 | 0.223 | 0.314 |
| Harmonic geodesic distance (HD) | 1.891 | 4.531 | 4.482 | 3.18 |
| Maximal degree | 4 | 16 | 14 | 9 |
| Nodes with max degree | OTU21193;  OTU12835;  OTU18393;  OTU18376;  OTU15304 | OTU17664 | OTU18499 | OTU13165 |
| Centralization of degree (CD) | 0.021 | 0.045 | 0.053 | 0.047 |
| Maximal betweenness | 85 | 5759.607 | 2928.471 | 710.424 |
| Nodes with max betweenness | OTU15304 | OTU17005 | OTU18198 | OTU13165 |
| Centralization of betweenness (CB) | 0.01 | 0.124 | 0.112 | 0.057 |
| Maximal stress centrality | 85 | 23219 | 7261 | 1563 |
| Nodes with max stress centrality | OTU15304 | OTU12959 | OTU18198 | OTU13165 |
| Centralization of stress centrality (CS) | 0.01 | 0.5 | 0.279 | 0.125 |
| Maximal eigenvector centrality | 0.538 | 0.358 | 0.377 | 0.486 |
| Nodes with max eigenvector centrality | OTU15304 | OTU17664 | OTU18499 | OTU13165 |
| Centralization of eigenvector centrality (CE) | 0.518 | 0.335 | 0.355 | 0.46 |
| Density (D) | 0.011 | 0.009 | 0.011 | 0.012 |
| Reciprocity | 1 | 1 | 1 | 1 |
| Transitivity (Trans) | 0.148 | 0.179 | 0.258 | 0.229 |
| Connectedness (Con) | 0.033 | 0.565 | 0.444 | 0.128 |
| Efficiency | 0.807 | 0.989 | 0.984 | 0.944 |
| Hierarchy | 0 | 0 | 0 | 0 |
| Lubness | 1 | 1 | 1 | 1 |

Table S3 The characteristics of empirical fungal network structure constructed under different planting years

| **Network Indexes** | **MW** | **5 Years** | **10 Years** | **20 Years** |
| --- | --- | --- | --- | --- |
| Total nodes | 220 | 114 | 102 | 96 |
| Total links | 1215 | 343 | 303 | 211 |
| R square of power-law | 0.762 | 0.791 | 0.815 | 0.733 |
| Average degree (avgK) | 11.045 | 6.018 | 5.941 | 4.396 |
| Average clustering coefficient (avgCC) | 0.292 | 0.319 | 0.286 | 0.245 |
| Average path distance (GD) | 3.081 | 3.264 | 3.561 | 3.775 |
| Geodesic efficiency (E) | 0.383 | 0.368 | 0.353 | 0.326 |
| Harmonic geodesic distance (HD) | 2.612 | 2.717 | 2.834 | 3.071 |
| Maximal degree | 45 | 30 | 21 | 17 |
| Nodes with max degree | OTU120 | OTU95 | OTU1643 | OTU1643 |
| Centralization of degree (CD) | 0.156 | 0.216 | 0.152 | 0.135 |
| Maximal betweenness | 1607.57 | 883.839 | 676.656 | 887.168 |
| Nodes with max betweenness | OTU120 | OTU473 | OTU642 | OTU1643 |
| Centralization of betweenness (CB) | 0.059 | 0.125 | 0.11 | 0.175 |
| Maximal stress centrality | 12562 | 5084 | 2614 | 3045 |
| Nodes with max stress centrality | OTU120 | OTU473 | OTU297 | OTU1643 |
| Centralization of stress centrality (CS) | 0.456 | 0.722 | 0.414 | 0.6 |
| Maximal eigenvector centrality | 0.219 | 0.342 | 0.293 | 0.378 |
| Nodes with max eigenvector centrality | OTU118 | OTU95 | OTU1643 | OTU1643 |
| Centralization of eigenvector centrality (CE) | 0.175 | 0.293 | 0.233 | 0.323 |
| Density (D) | 0.05 | 0.053 | 0.059 | 0.046 |
| Reciprocity | 1 | 1 | 1 | 1 |
| Transitivity (Trans) | 0.371 | 0.432 | 0.371 | 0.351 |
| Connectedness (Con) | 0.903 | 0.803 | 0.961 | 0.879 |
| Efficiency | 0.949 | 0.944 | 0.948 | 0.958 |
| Hierarchy | 0 | 0 | 0 | 0 |
| Lubness | 1 | 1 | 1 | 1 |

**
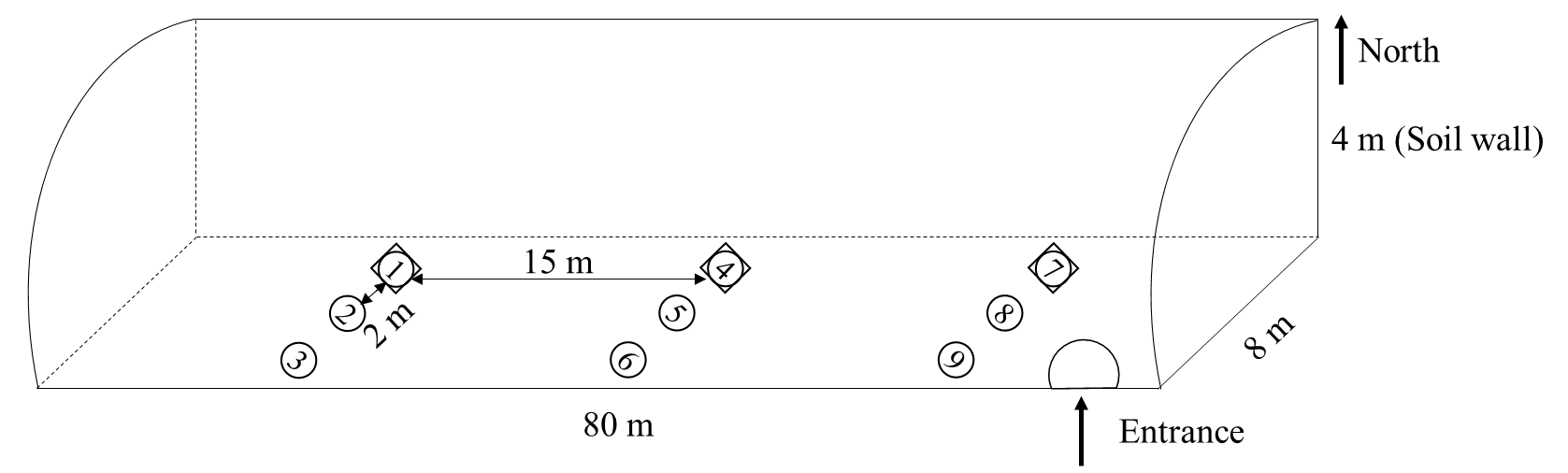
**

**Fig. S1** Schematic diagram showing the soil sampling positions in a greenhouse (n = 3). The soil samples from sampling point 1 to 3 were mixed together as a replication, 4 to 6 and 7-9 were separately mixed as other two replications.


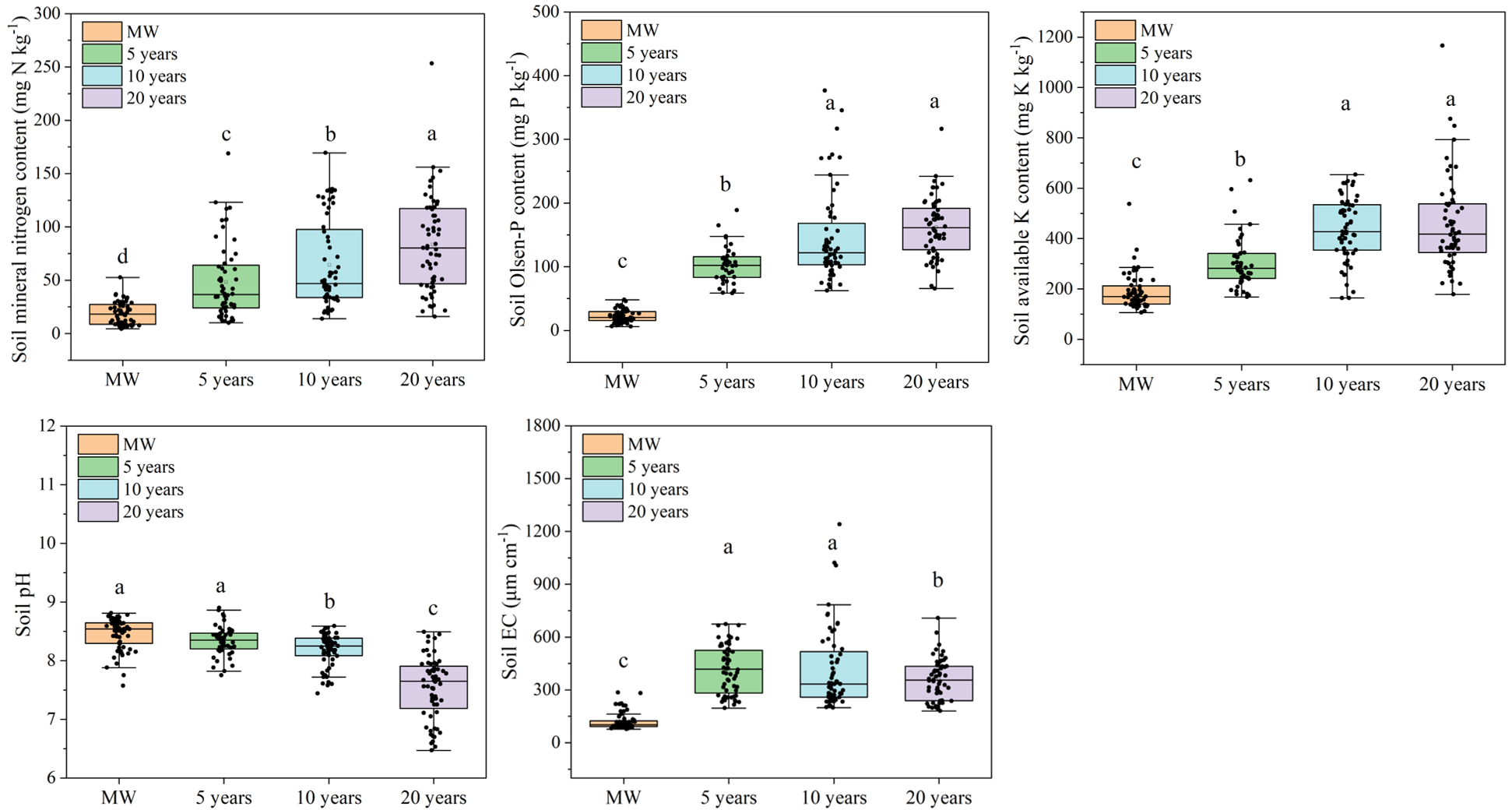


**Fig. S2** Soil properties at the 0-30 cm depth in tomato fields with varying years of cultivation, as well as in adjacent maize and wheat fields (MW). The different lowercase letters represent significant differences (*p* < 0.05) between different planting years. The boundaries of the boxes indicate the first and third quartiles; the lines and squares within the boxes represent the medians. Whiskers mark the 10% and 90% percentiles, and the outliers are shown as dots. The error bars represent the standard error of the mean.

**
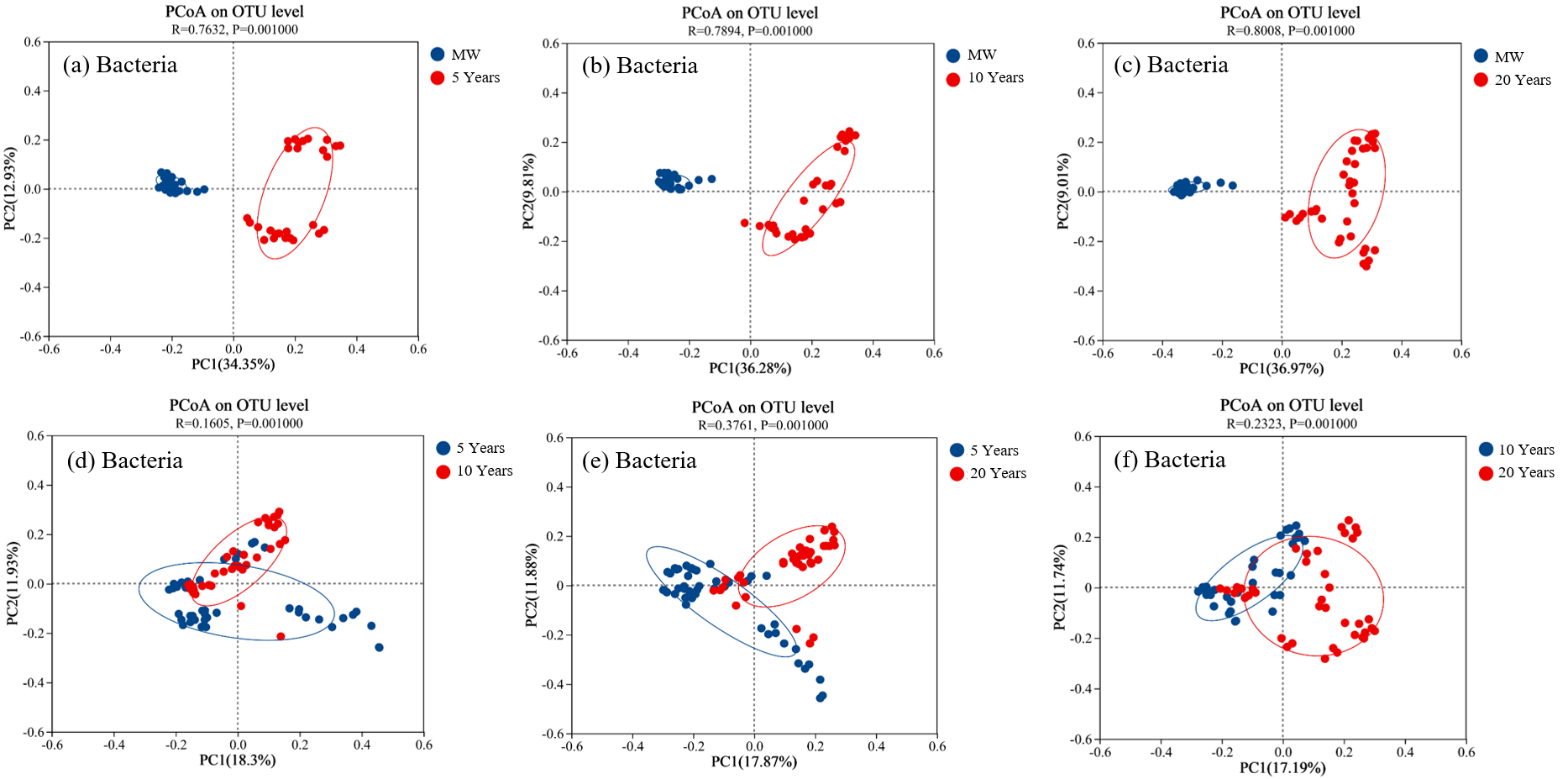
**

**Fig. S3** Principal coordinate analysis of the bacterial communities between any two planting years (n =27).


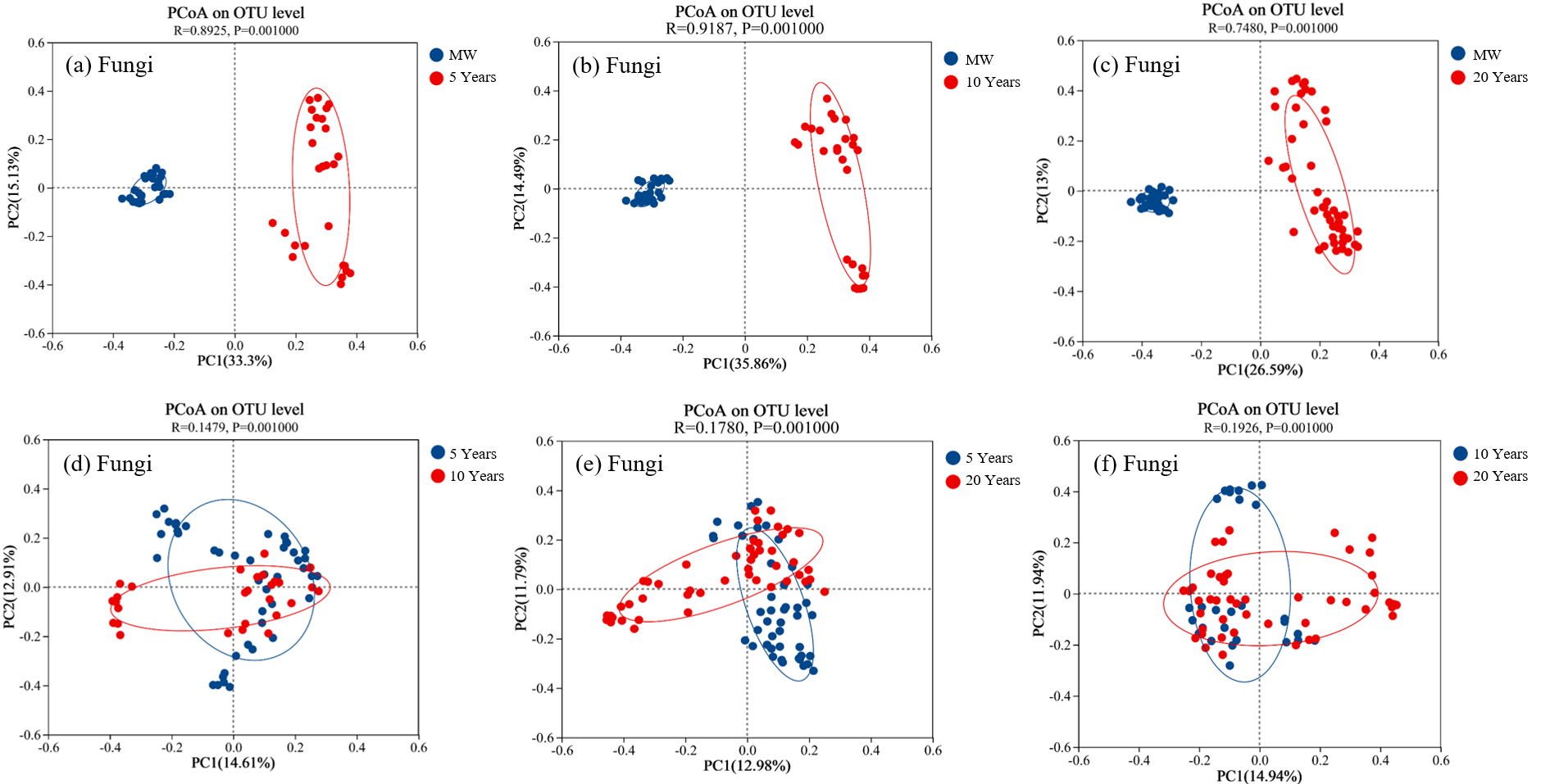


**Fig. S4** Principal coordinate analysis of the fungal communities between any two planting years (n =27).


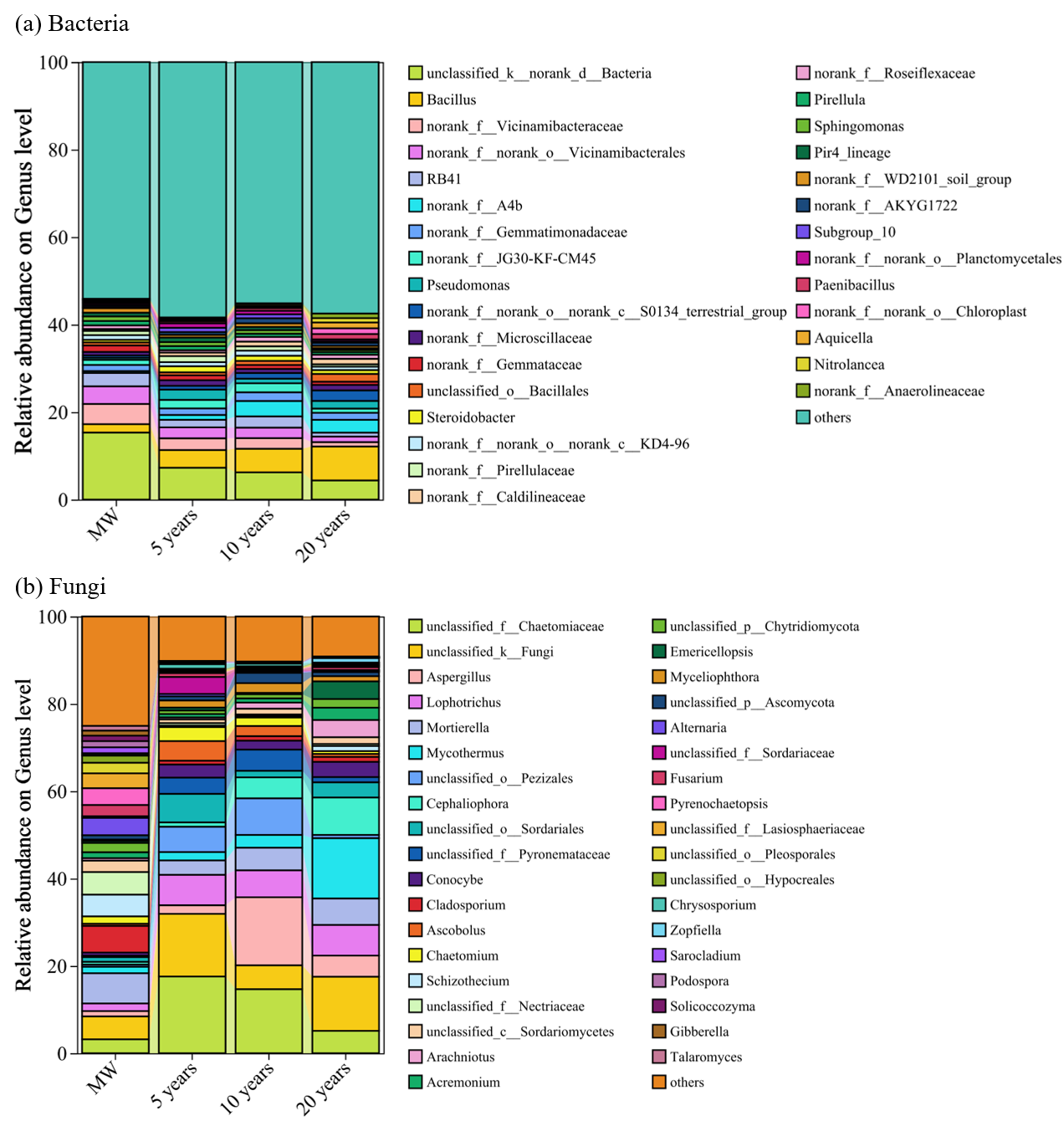


**Fig. S5** Soil bacterial (a) and fungal (b) community bar plot analysis under different planting durations at the genus level (n=27). Others = the relative abundance was ≤1%.


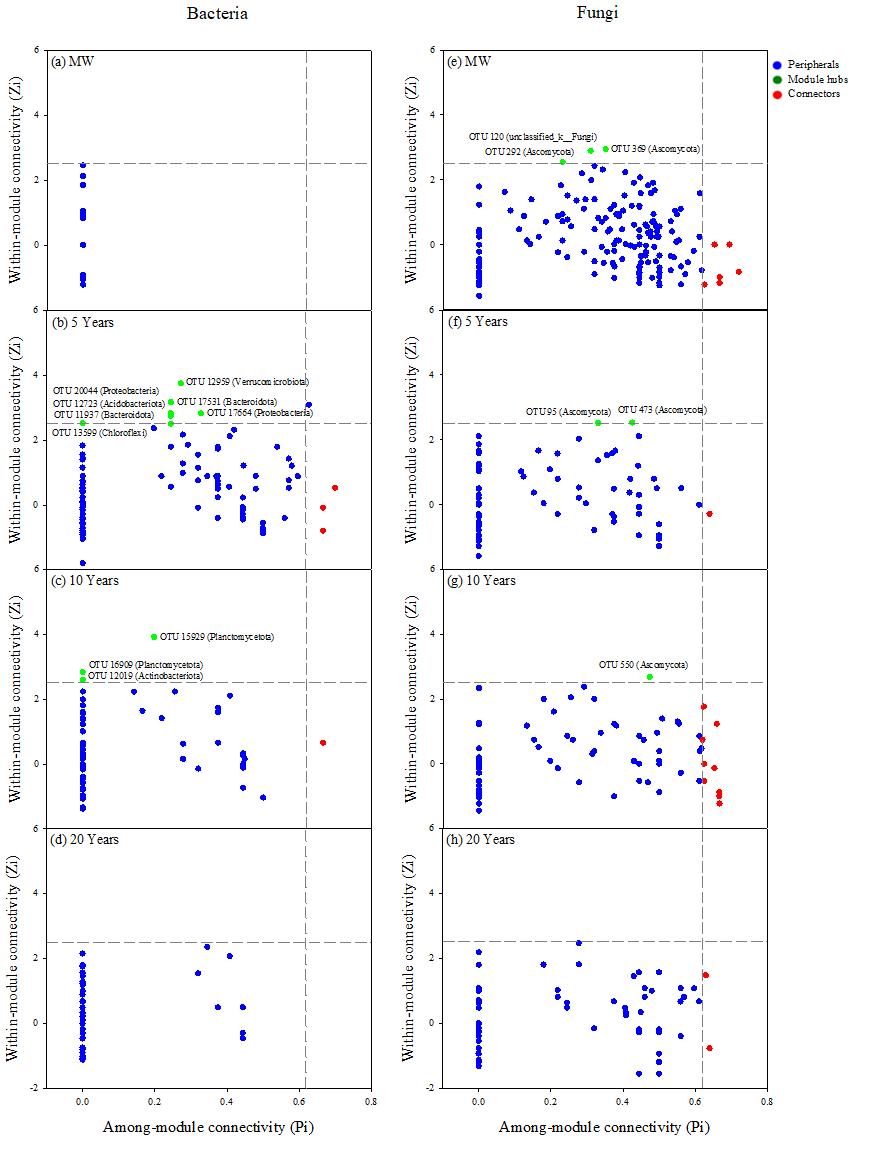


**Fig. S6** Z‒P plot showing the distribution of OTUs on the basis of topological role under different planting years (a-d: bacteria; e-h: fungi). Each point represents an OTU. The location of each OTU was determined according to the within-module connectivity (Zi) and among-module connectivity (Pi). The module hub is defined according to the Zi and Pi values (Zi>2.5, Pi≤0.62). The four identified module hubs are marked with OTU numbers and their phyla.
